# On-the-fly investigation of XUV excited large molecular ions using a high harmonic generation light source

**DOI:** 10.1038/s41598-022-17416-4

**Published:** 2022-08-01

**Authors:** Marius Hervé, Alexie Boyer, Richard Brédy, Abdul-Rahman Allouche, Isabelle Compagnon, Franck Lépine

**Affiliations:** Univ Lyon, Université Claude Bernard Lyon 1, CNRS, Institut Lumière Matière (UMR 5306 CNRS), 10 rue Ada Byron, Campus Lyon Tech La Doua, 69622 Villeurbanne Cedex, France

**Keywords:** Atomic and molecular interactions with photons, Attosecond science, High-harmonic generation

## Abstract

We present experiments where extreme ultraviolet femtosecond light pulses are used to photoexcite large molecular ions at high internal energy. This is done by combining an electrospray ionization source and a mass spectrometer with a pulsed light source based on high harmonic generation. This allows one to study the interaction between high energy photons and mass selected ions in conditions that are accessible on large-scale facilities. We show that even without an ion trapping device, systems as large as a protein can be studied. We observe light induced dissociative ionization and proton migration in model systems such as reserpine, insulin and cytochrome c. These results offer new perspectives to perform time-resolved experiments with ultrashort pulses at the heart of the emerging field of attosecond chemistry.

## Introduction

Interaction of light with gas phase molecules has been used for decades to study the properties of isolated systems as well as the out of equilibrium processes involved in such interaction. To study the dynamics of gas phase systems that can occur on timescales ranging from attosecond to minutes, a variety of experimental technics have been developed using light sources covering a wide spectral range from far-infrared up to hard X-ray. With the recent advances in light sources, new perspectives and challenges have been identified, such as the access of attosecond dynamics of electrons, the observation of structural changes in complex molecules or the understanding of electron correlation effects^1,2^.

The investigation of attosecond timescale dynamics has become accessible for small quantum systems or small molecules by using table top light sources based on high harmonic generation (HHG)^3^. HHG is a highly nonlinear process that converts the fundamental frequency of intense femtosecond laser into high order harmonics which can extend from extreme ultraviolet (XUV) up to soft-X-ray regime^4^, generating light pulses of attosecond duration. These advanced technologies are so far limited to the study of molecules that can be stored in gas bottle or evaporated using ovens^5–7^, but are not yet available when one considers much larger and fragile species, such as proteins.

When considering large molecular species, electrospray ionization (ESI) is the state-of-the-art technics to transfer intact, fragile and large ions from solution to the gas phase. With this technics, a huge variety of complex molecular ions of different charge states can be injected into vacuum, from small molecules up to macromolecules. A precursor ion of given mass over charge (m/z) can then be selected and usually stored in ion trapping or storing device to increase the density of the ion cloud, allowing for further investigation. While ion-trapping devices bring advantages in terms of mass spectrometry analysis technics, the existing instruments do not allow, for example, detection of emitted electrons, thus limiting the exploitation of electron based spectroscopic technics or time resolved crossed-beams type experiments^2^.

Nevertheless, tremendous progress have been made in the spectroscopy of large ions. Experiments have been developed over a broad range of electromagnetic radiations, from GHz up to soft X-ray domains in order to elucidate molecular structures and electronic properties by performing rotational^8^, vibrational^9^, electronic^10–12^ or innershell^13^ action spectroscopy. The use of light in the IR, UV–Vis or UV domain as an activation method to generate efficient fragmentation has been thoroughly investigated over the past 20 years^12,14,15^, enabling the characterization of large molecular structures. These approaches were developed as complementary methods to overcome the limitations of collision induced dissociation (CID) that is based on collision with neutral gas and therefore driven by energetic barriers and statistical laws^16–19^. Recently, activation with ultrashort femtosecond laser pulses has proved its potential by unveiling specific fragments following ionization of protonated biomolecules^20–23^.

Extending these approaches to higher photon energy, large scale facilities have also been used to provide new activation methods. At synchrotron light-sources, activation of protonated and deprotonated proteins were performed using photon energies in the vacuum ultraviolet (VUV) range of 5–20 eV. By making use of efficient valence ionization, VUV excitation provides new tools to create reactive radicals and new fragmentation pathways^13,24,25^. Synchrotron can also deliver X-ray radiation that provides local excitation with site selectivity inducing new photoinduced reaction as demonstrated in peptides^26,27^. With the emergence of new large scale facilities such as free electron lasers (FEL), high energy photon at high flux and with short pulse duration are now available, offering new opportunities in terms of sensitivity, and potential applications in terms of non-linear interaction^28^. In pioneer experiments, Schlathölter and co-workers demonstrated the interest of protein activation in the gas phase using FEL pulses by showing that ubiquitin responds as an ensemble of small peptides^29^. Although offering new exciting possibilities, the use of large scale facilities brings additional constraints, incompatible with daily use for analytical purposes or laboratory studies^30^. New approaches have been proposed using low cost, high photon energy sources. Giuliani et al.^31^ demonstrated the use of discharge lamp that delivers a continuum flux of photons in the range of 15–30 eV and coupled to tandem mass spectrometry for molecular activation^32^. Even though the cited experiments have provided an enormous amount of information on large molecular ions, no experiment has been able to measure realtime ultrafast processes involved in high energy photon excitation. A step in the development of experiments able to measure ultrafast processes in large ions without using trapping devices was made in a recent time-resolved experiments using “on-the-fly” configurations^20,33^. This has allowed the investigation of ultrafast processes using UV–visible laser pulses^34^. The development of experiments combining short XUV light pulse technology and ESI devices is therefore a necessity to push forward the studies on the dynamics of large molecules but remains challenging.

Here we used a combination of high harmonic generation XUV source with electro-spray ionization source and mass spectrometry (MS) technics, to study the interaction between an ultrashort XUV pulse and complex molecular ions. The HHG source uses an intense femtosecond laser to generate photons with energy in the range of 10–50 eV confined in ultrashort 20 fs pulses at a 5 kHz repetition rate. The mass spectrometer does not include any trapping device. It rather uses an “on-the-fly” configuration in which each ion interact only once with the light pulse as in crossed beam experiments. We investigated photoinduced reactions in reserpine and two proteins (cytochrome c and insulin). We observed that XUV excitation of complex protonated molecules leads to ionization followed by proton migration and dissociation. These experiments demonstrate the feasibility of ultrafast experiments in complex molecular ions.

## Methods and materials

### XUV–ESI–MS instrument

**Figure 1:**
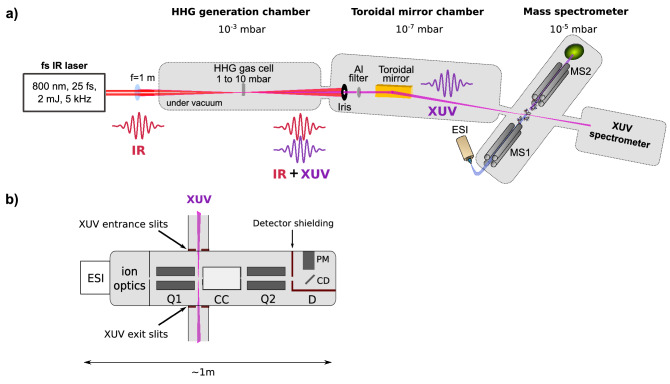
Experimental set-up XUV–ESI–MS. (**a**) A beam of XUV pulses is created by high harmonic generation using an infrared (IR) femtosecond laser focused in a gas cell. The residual IR beam is blocked by an iris and a thin aluminum filter that also eliminates low order harmonic. The XUV beam is focused in a mass spectrometer with a toroidal mirror and characterized using a standard XUV spectrometer. (**b**) The mass spectrometer is a modified Xevo-TQS-micro from Waters. It is equipped with an electrospray ionization source (ESI), includes two linear quadrupole mass filters, one for selection of the precursor ions (Q1, MS1) and one for the analysis of the charged products (Q2, MS2), a collision cell (CC) and a detector (D) based on a conversion dynode (CD) and a photomultiplier (PM). The interaction region is located within an rf only ion guide (post-filter, not represented) between Q1 and CC. The interaction of a XUV pulse and molecular ions occurs without trapping device. This is referred to as “on-the-fly’ configuration.

Our instrument combines an electro-spray source, a mass selector, a mass spectrometer and a pulsed XUV femtosecond source. A schematic of the instrument is presented in Fig. 1. Figure 1a presents the main parts of the XUV beamline with a simplified schematics of the mass spectrometer. Figure 1b shows a sketch of the mass spectrometer with the main modifications for its coupling to the XUV beamline.

### XUV light source

**Figure 2:**
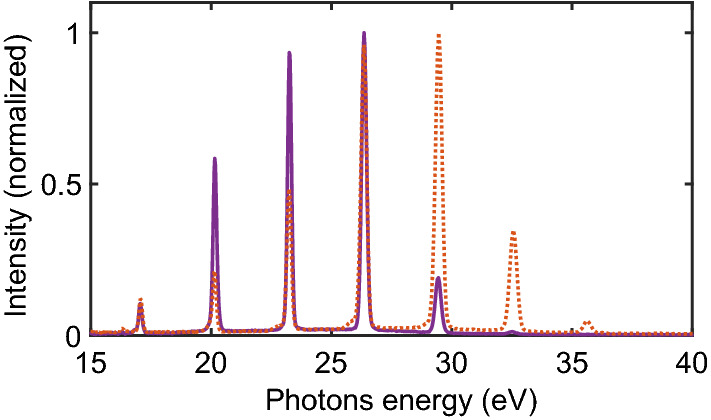
XUV photon spectrum generated using xenon (purple full line) and krypton (orange dashed line) in the HHG cell and with aluminum filter.

The light source is designed to fit the required performances of experiments where low-density targets interact with the light delivered by a low photon flux source. The source is based on a commercial femtosecond laser system from Coherent delivering IR (800 nm), 2 mJ, 25 fs pulses at 5 kHz repetition rate. The IR beam is used to generate XUV radiation by high harmonic generation (HHG) with a flux of 10^12^ photons per second. HHG is a highly nonlinear process that converts IR fundamental light into high order harmonics. To do so, the IR beam is focused with a 1 m focal lens onto a 4 mm long rare gas cell leading to a focal spot estimated at 130 µm. The IR light is divergent and is mostly blocked by an iris located at 80 cm after the cell. The generated XUV light is weakly divergent and therefore can be transmitted through the hole of the iris. A metallic filter is located 2 cm after the iris, which allows eliminating the remaining IR light as well as low order harmonics. Depending on the chosen metal filter the transmitted XUV spectrum can be tuned from 10 to 50 eV. In the case of an aluminum filter of 200 nm thickness, harmonics below 17 eV are filtered out. After the filter, the XUV light is focused by a 1 m focal length toroidal mirror onto the interaction region of the mass spectrometer described in the next section. The focal spot of the XUV beam in the interaction region is estimated at about 100 μm. After the mass spectrometer, a standard XUV spectrometer allows to measure the XUV spectrum generated. Figure 2 presents a typical XUV spectrum in the range of 15 to 35 eV generated using xenon or krypton gas in the generation cell.

The long focal configuration is necessary to insure high photon flux required with this instrument. However, it also introduces intrinsic instabilities compared to tighter focusing configurations. Overall, the high stability required for long acquisition time necessitates a compromise between focal, flux and stability.

### ESI mass spectrometer

The XUV source is combined with a triple quadrupole mass spectrometer, a Xevo TQ-S-micro provided by Waters. While this instrument was designed for analytical purpose based on CID experiments, we have modified it to allow for laser interaction^20,33^. The mass spectrometer consists of an electrospray ionization source, ion optics, two linear quadrupole mass filters, a collision cell and a detection device. Molecules in liquid sample are injected at a flow rate of 300 μL/h through a micrometer capillary tube at a voltage of about 3 kV. The charged droplets generated at the exit of the capillary tip are gradually transferred under a nitrogen gas flow from the atmosphere to the vacuum chambers of the spectrometer. The droplets are evaporated by the action of the gas, voltage and electrostatic repulsion; eventually leading to isolated charged molecular ions. The solvent is therefore progressively eliminated and pumped in a differentially pumped chamber. The free jet of molecular ions evolves then in a vacuum chamber at an overall pressure of 1 × 10^−5^ mbar. Ions are manipulated using quadrupole mass filters and experiments are performed using MS–MS operating mode. The precursor ion of interest is mass and charge (m/z) selected (MS1) with a first quadrupole (Q1). After interaction, the product ions are m/z analyzed (MS2) by a second quadrupole (Q2) before their detection. The detection device includes a conversion dynode and a phosphor screen to convert the ionic signal into photons that are then detected by a photomultipler. The instrument is equipped with a traveling wave collision cell^35^ between Q1 and Q2 for CID experiments. The collision cell is filled with argon gas at a pressure of 3.5 × 10^−3^ mbar to ensure high transmission through the cell and collision energy voltage can be tuned from 0 to 120 V. The instrument can detect ions in the mass range 2 to 2048 m/z with a mass resolution of 4000 and achieve acquisition rates up to 20,000 Da/s. The acquisition of a mass spectrum is performed using MassLynx™ software from Waters. A mass spectrum results in the integration of several scans of a specified mass range. In this work, the mass range is scanned by the quadrupole Q2 in typically 0.5–5 s (scan time) and a mass spectrum is typically acquired in 5 min (acquisition time). We set the scan time and acquisition time in order to achieve sufficient statistics and resolution for the chosen mass range.

### XUV–ESI–MS coupling

The spectrometer had been modified in order to excite ions with photons^20,33^. Window ports have been drilled to allow for the entrance and exit of the laser beam. For XUV/HHG light interaction, the mass spectrometer (10^−5^ mbar) is connected to the toroidal mirror chamber (10^−7^ mbar) of the photon beamline by a pipe of diameter 25 mm and about 30 cm long that holds the pressure gradient. This is required to ensure propagation of the XUV light without absorption along the path. Because of the high sensitivity of the photomultiplier it is crucial to protect the transport of the light into the instrument. This was done by adding slits at the entrance and exit ports of the instrument in order to minimize the noise induced by scattered light. The detection device has been shielded and filters installed to further reduce detection of scattered light within the instrument. The interaction region is located at the exit of Q1, before the collision cell. The XUV light is focused between the rods of a short quadrupole (post-filter) acting as a rf-only ion guide between Q1 and the collision cell. The interaction region, delimited by the intersection of the XUV focal point (diameter about 100 μm) and the ion beam (diameter about 1 mm), is located about 1 cm before the entrance hole (2 mm in diameter) of the collision cell. The effective pressure in the interaction region results from a gradient of pressure between the collision cell (10^−3^ mbar) and the overall pressure around the interaction region (10^−5^ mbar) and depends on the effusion of the argon gas through the entrance hole of the collision cell. As typical kinetic energies of the ions in the spectrometer are in the order of the eV or few tens of eV, ions are travelling through the instrument at a velocity of several hundred meters per second. The velocity of ions with nearby m/z values and kinetic energy is practically the same. Therefore, ions are traveling several millimeters between two XUV pulses separated in time by 200 μs. This “on-the-fly” type of experiment ensures that ions interact only once with a pulse light, disentangling this interaction from “multiple-pulses” interactions. The “on-the-fly” configuration also offers the possibility to perform time resolved experiments^34^ or to measure, in principle, quantities difficult to access using traps such as electron emission or kinetic energy release. For photon interaction experiments collision voltage is set to 0 V to reduce interplay between the XUV induced signal and the residual CID background signal. Under these conditions, the travelling-wave collision cell acts as an rf-only ion guide toward Q2. The signal of the m/z ion of interest is optimized by adjusting the concentration of the ESI solution as well as the parameters of the spectrometer. Depending on the m/z selected ion, the ion current is estimated between 10^7^ and 10^9^ ion/s (1 to 100 pA for protonated molecules) which corresponds to a density of about 10–10^3^ ions/mm^3^ in the interaction region. We note that the continuous ion beam is irradiated only during a fraction of time by the XUV light (25 fs pulse every 200 μs). In the experiment, typically few ions, to few tens ions per second are excited by the XUV radiation. This contributes to the low signal-to-noise ratio in the experiment. The mass spectra are therefore mostly dominated by the intact precursor ion peak and residual CID product ions. Mass spectra are recorded with and without XUV light interaction. Without XUV light (laser OFF) the mass spectra correspond to the ones induced by the residual CID in the collision cell with 0 V collision voltage. As presented in the next section for protonated reserpine, cytochrome-c and insulin, it is noticeable that despite the very low duty cycle, product ions induced by the XUV light (laser ON) can be detected. This suggests a high efficiency of the XUV activation processes. The results demonstrate the capabilities of the instrument to study XUV induced processes in molecular ions.

### Chemicals and sample preparation

Reserpine (from Alfa Aesar L03506), human insulin (from Sigma-Aldrich I0908) and cytochrome c from bovin heart (from Sigma-Aldrich C2037) samples were prepared by dissolution in 50:50 MeOH:H_2_0 solution with 0.1% of acetic acid at a concentration of 60 μM for reserpine, 8 μM for insulin and 4 μM for cytochrome c.

## Results and discussion

### Reserpine

**Figure 3:**
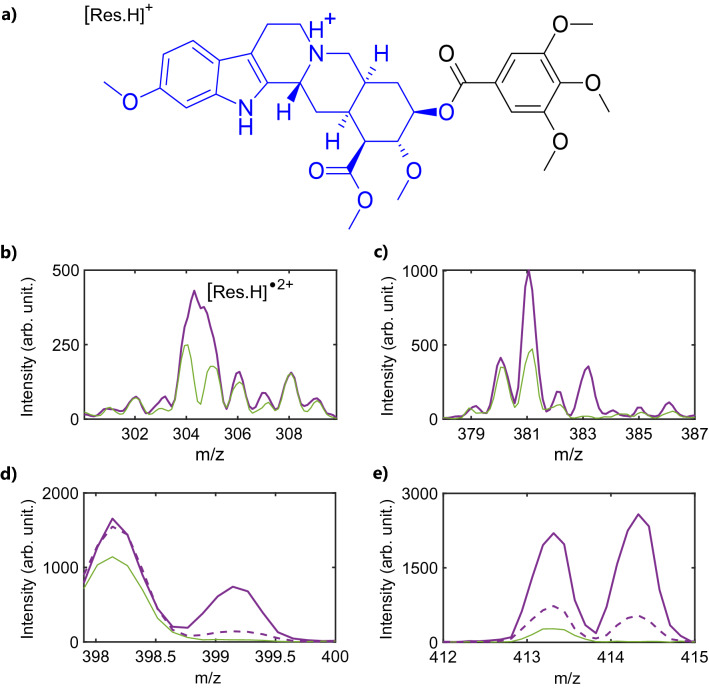
XUV induced ionization and dissociative ionization of reserpine. (**a**) scheme of protonated reserpine [Res.H]^+^ (m/z 609) with the methoxy indole based group (m/z 414) colored in blue and the trimethoxybenzoyl group (m/z 195) in black. (**b**)–(**e**) Mass spectra showing regions of interest for XUV (laser ON, purple) and residual CID (laser OFF, green) induced protonated reserpine signal around (**b**) m/z 304.5 ([Res.H]^•2+^ doubly charged reserpine), (**c**) m/z 381–383, (**d**) m/z 399 and (**e**) m/z 413–414. (**b**)–(**e**) XUV is generated using xenon in the HHG gas cell (full line). Dashed line in (**d**)–(**e**): krypton gas is used for HHG instead of xenon, peak intensities normalized using CID signal.

Protonated reserpine [Res.H]^+^ (m/z 609, Fig. 3a) is a model molecule used as a standard in mass spectrometry but also for pharmaceutical purpose^36,37^. With a pentacyclic core, the molecule can be seen as a trimethoxybenzoyl group (TMB, m/z 195) bonded to a methoxy indole based group (m/z 414). Figure 3b–e show the mass spectra regions of interest resulting from the interaction between protonated reserpine and the XUV light generated with xenon gas (full purple line, laser ON) compared with the residual CID experiment (full green line, laser OFF). Fragments observed in the CID mass spectrum correspond to those observed in the literature^20,36^.

While common fragments are observed with both activation methods, specific fragmentation pathways are observed in the case of XUV interaction as shown in Fig. 3. The ionization potential of the protonated reserpine is expected to lie slightly above the neutral molecule (IP_neutral_ = 7.88 eV^38^ due to Coulombic effect of the added proton and/or structure modification^39^. With XUV photon energies in the range 15–35 eV ionization is expected for molecule such as reserpine and may result in specific, non-statistical, fragmentation. Fragments at m/z 413 and 414 correspond to reserpine after a loss of TMB (m/z 195) with or without proton. As fragment m/z 414 is not observed in CID spectra, one might assume that the XUV induced fragments m/z 414 and 413 result from dissociative ionization of the protonated reserpine. This is consistent with the observation of a peak at m/z 304.5 corresponding to the doubly charged protonated reserpine [Res.H]^•2+^ following XUV excitation (Fig. 3b) (due to the instrument mass resolution the XUV induced doubly charge peak is overlapping with the neighbor masses at m/z 304 and 305 leading to a broad feature). We note that these fragments are also present in activation experiments with intense 800 nm femtosecond laser pulse^20^. Additional fragments are observed at m/z 398 and 399, which correspond to the reserpine after TMB (m/z 195) plus oxygen loss and hydrogen migration. Fragments at m/z 381–383 corresponds to the reserpine after TMB (m/z 195), plus oxygen and CH_3_ or CH_4_ loss and hydrogen migration. These fragments may result from sequential dissociation following ionization, indicating that enough internal energy remains after ionization.

Using krypton gas for HHG instead of xenon results in higher photon energies while the photon flux is lower. The same fragmentation channels are observed with different relative intensities as depicted in Fig. 3 for fragments m/z 413–414 and 398–399 (dashed line). Comparing the peaks at m/z 414 and 399 in Fig. 3, one can notice that both intensities drop compared to fragments m/z 413 and 398 in the case of fragmentation induced by XUV generated with krypton compared to xenon. This illustrates the tunable capacity of the HHG source used to excite different electronic states resulting in different fragmentation schemes or branching ratios.

### Quantum chemistry calculations

**Figure 4:**
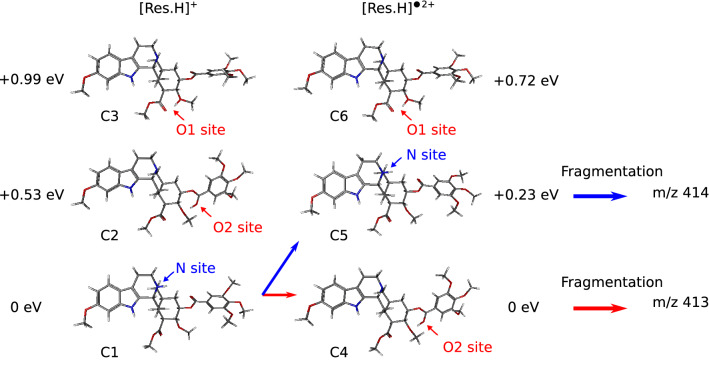
Protonation sites of reserpine. C1, C2, C3: most stables configurations for [Res.H]^+^, the proton is located respectively near the N, O1 and O2 sites. C4, C5, C6: most stables configurations for [Res.H]^•2+^, the proton is located respectively near the O2, N and O1 sites. XUV photoionization of C1 can lead to fragment m/z 413 or 414 depending on the populated configuration C4 or C5. The [Res.H]^•2+^ ground state C4 lies about 8.92 eV above C1.

The O–R bond cleavage after the carbonyl group in the ester bridge of the protonated reserpine leads to fragment m/z 414 while fragment m/z 413 results from the same bond cleavage but with an additional hydrogen migration to the carbonyl moiety. To validate this interpretation, quantum chemistry calculations were performed using density functional theory. Structures of protonated reserpine [Res.H]^+^ and ionized protonated reserpine [Res.H]^•2+^ in the ground state were optimized using CAM-B3LYP method, with 6-311++G** basis set. The calculations, performed using Gaussian^40^ and Gabedit^41^, show three possible protonation sites (Fig. 4). For [Res.H]^+^ the lowest energy geometry (C1) corresponds to the presumed protonation site with the proton bonded to the nitrogen at the tertiary amine^42^, as depicted in Figs. 3a and 4. For the two other configurations the proton is bonded either near the oxygen atoms of the TMB group (O2 site, see C2 structure in Fig. 4) or near the oxygen of the methoxy indole based group (O1 site, see C3 structure in Fig. 4). For [Res.H]^+^ these two conformations lie about 0.5 eV (C2, 0.5299 eV above C1) and about 1 eV (C3, 0.9991 eV above C1) above the lowest energy conformation. From these calculations, one can assume that only the C1 geometry, with a localized proton at the nitrogen site, has to be considered in this experiment. When the molecule is ionized, two charges (one proton, one hole) are carried by the photoproduct. The location of these two charges depends on both electrostatic repulsion and global interaction with the surrounding atoms. For [Res.H]^•2+^ the lowest energy conformation corresponds to protonation near the oxygen of the TMB group (O2 site, C4 structure, 8.9175 eV above C1) and the conformation C5 with the proton bonded to the nitrogen at the tertiary amine lies 0.23 eV above it (9.1518 eV above C1). The energy of the structure C6 (9.6397 eV above C1) with the proton at the O1 site is 0.72 eV above the ground state geometry C4. As ionization is a fast process compared to proton migration, the energy difference between C5 and C1 conformations gives an estimate of about 9.1 eV for the calculated vertical ionization potential of the protonated reserpine. These calculations indicate that ionization of the protonated reserpine in the ground state may trigger a proton migration from the N site to the O2 site. In this work the ionized protonated reserpine [Res.H]^•2+^ results from XUV excitation and large amount of internal energy can remain in the molecule after ionization. Due to the low energy gap (0.23 eV) between the O2 and N site structures, these two conformations of the molecule can be populated after XUV photoionization. Their dissociation can result in the observation of the fragment m/z 414 if the proton is initially localized on the N site (population of conformer C5) and of the fragment m/z 413 in the case of the O2 protonation (population of conformer C4). This shows that starting with a stable molecule with a localized proton, the XUV excitation leads to an ionized protonated specie that can be found in the two configurations due to proton migration with fragmentation products that depend on the proton localization.

This first example demonstrates the interaction between the XUV radiation and the protonated reserpine molecule. The excitation induces an efficient ionization of the molecule accompanied by sequential dissociation and proton migration.

### Proteins: insulin and cytochrome c

In the following, we present the results of the interaction of XUV photons of 15–30 eV with two proteins of different size, structure and composition: human insulin (mass 5808 Da) and cytochrome c from bovine heart (mass 12,327 Da).

The human insulin is a protein composed of 51 amino acids organized in two peptide chains linked by disulfide bonds. Ionization and fragmentation of insulin in various charge states have been studied using several activation methods to investigate, for example, protein disulfide bonds cleavage^43–46^. Single and double ionization, non-dissociative ionization as well as emission of fragments from amino acid have been observed in X-ray excitation of [Ins.5H]^5+^. Sequence ions from the N-terminals of both chains were also observed^44^.

The cytochrome c protein is composed of 104 amino acids surrounding a heme molecule that is a porphyrin with a central iron atoms and that is covalently bond to the peptide chain. The mass of the protein is about 12 kDa and depends on the amino acid sequence that varies with the species. Multiply protonated cytochrome c had been studied in several gas phase experiments using VUV photons (< 20 eV)^24,39,47^ and soft X-rays (> 280 eV)^44,48^. Experiments in the intermediate photon energy range has not been reported so far. However, we note that this intermediate energy range was used by Schlathölter et al. for studying other large species, such as ubiquitin (8.5 kDa), or peptides of mass below 3 kDa^29,49,50^.

**Figure 5:**
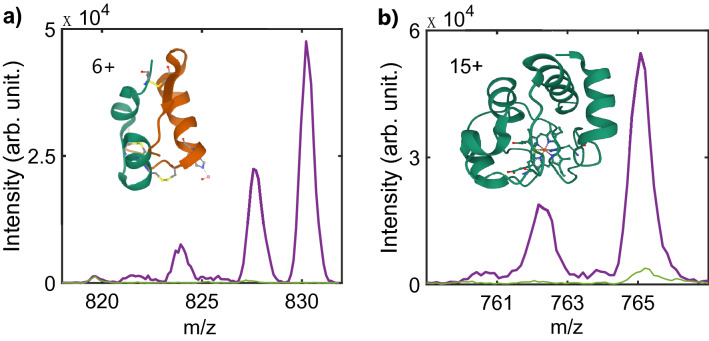
XUV induced ionization and dissociative ionization of insulin and cytochrome c proteins (laser ON, purple) compared with residual CID (laser OFF, green). (**a**) Mass spectrum in the range m/z 818–832 for insulin precursor [Ins.6H]^6+^ (m/z 969). The ionized protein [Ins.6H]^•7+^ is observed at m/z 830. Dissociative ionization with loss of neutral fragment is observed at m/z 828, 824. Inset: crystal structure from the RCSB protein data bank (PDB ID 4RXW). (**b**) Mass spectrum in the range m/z 700–770 obtained for precursor [Cyt-c.15H]^15+^ (m/z 816). Peaks corresponding to the ionized protein ([Cyt-c.15H]^•16+^, m/z 765) and dissociative ionization by neutral loss is observed (m/z ~ 762: loss of CO_2_ or acetyl groups C_2_H_3_O). Inset: crystal structure from the RCSB protein data bank (PDB ID 2B4Z).

In this work, only multiply charged protonated species corresponding to m/z below 2 kDa can be observed within the mass range of our spectrometer. We focus our study on the six times protonated insulin [Ins.6H]^6+^ (m/z 969) and a fifteen times charged cytochrome c [Cyt-c.15H]^15+^ (m/z 816). A representation of the crystal structure of these proteins from the RCSB protein data bank (PDB)^51,52^ is depicted on Fig. 5 (PBD ID: 4RXW for insulin and 2B4Z for cytochrome c). Figure 5 shows the most striking signature of the interaction between the proteins with the XUV light. The XUV induced signal (laser ON, purple) is compared with the residual CID (laser OFF, green).

For insulin [Ins.6H]^6+^ (Fig. 5a) the interaction with the XUV leads to specific masses observed at m/z 830, 828 and 824. The peak at m/z 830 corresponds to [Ins.6H]^•7+^. This is the parent protein after one electron loss. The peak observed at m/z 828 corresponds to the [Ins.6H]^•7+^ molecule after loss of NH_3_, OH or H_2_O groups. The fragment at m/z 824 also corresponds to dissociative photoionization of the protein after loss of a CO_2_ molecule. These channels are in line with the main ones observed in X-ray excitation^44^. The typical fragments obtained by CID have been identified in the literature^45,46^. These channels are very weakly present in our XUV induced experiment. It is also noticeable that our data show no evidence of cleavage of the disulfide linkages as observed in CID for [Ins.5H]^5+^.

For cytochrome c [Cyt-c.15H]^15+^, the XUV interaction leads to specific fragments between m/z 700 and m/z 770 that are very weakly or simply not present in the case of CID. The ion at m/z 765 (Fig. 5b) corresponds to the parent protein that has lost one electron due to valence photoionization [Cyt-c.15H]^•16+^. The ionization potential of the fifteen times protonated cytochrome c has been measured to about 14 eV using synchrotron radiation^39^, which is lower than the XUV photon energy used in our experiment, and makes photoionization a highly probable process. Other peaks are present at m/z < 765. These peaks correspond to the loss of small neutral fragments by the protein associated to loss of the lateral peptide chain, as well as loss of terminal ends of the chains. Although the mass resolution prevents a detailed identification of each individual fragmentation pathway, one can identify the loss of CO_2_ or acetyl groups (loss of a mass 44 Da, peak at m/z ~ 762), of the lateral chain of glutamic acid (loss of 73 Da, peak at m/z ~ 760); of the lateral chain of tyrosine (loss of 107 Da) and of the residue tryptophan at the C-terminal (loss of 130 Da). These losses are generally observed for ionized proteins^44,47,50^. For [Cyt-c.15H]^15+^, at this charge state, the native structure of the molecule is not preserved and the protein is expected to exist in a nearly linear extended conformation^53^. The fragmentation pattern, which depends on the structure and charge location, has been described in previous experiments reported in the literature. It corresponds to the dissociation of some of the amino acids constituting the molecule (peak ratio m/z < 200) and cleavages of the peptide chain^54,55^. In our experiment, these channels are much weaker than dissociative ionization of the protein. Double ionization ([Cyt-c.15H]^••17+^) is also observed around m/z 720. This is in line with the photon energy range used in this work and the evolution of the ionization potential of cytochrome c that presents nearly a plateau around 14 eV for charge between 12+ and 15+.

As a conclusion, we clearly observe an effective interaction between XUV photons and proteins, insulin and cytochrome c. The resulting photoionization and dissociative photoionization processes correspond to the efficient valence ionization of the protein, leaving the molecule in a higher charge state with a sufficient amount of internal energy to dissociate via neutral losses. Ionization of the residual argon gas in the interaction region can produce background electrons. Although it cannot be ruled out, we observed no sign of interaction between these electrons and the molecular ions. The observed dissociative ionization processes are specific to the interaction with high energy photons in agreement with synchrotron experiments. This illustrates that the XUV excitation does not only lead to ionization of the molecule, but that the remaining internal energy of the molecule is sufficient to trigger fragmentation. Open questions regarding the localization and/or redistribution of internal energy on such large molecules remain. These questions could be further investigated using time resolved experiments that are now in reach with our XUV–ESI–MS instrument. Remarkably, these results are obtained without any trapping device.

## Conclusion

We have performed experiments where mass selected molecular ions are excited using table top XUV sources based on HHG. This is done using an ESI–MS mass spectrometer coupled to an XUV source operating at 5 kHz. The interaction between the ions and the light occurs “on the fly”, meaning that no trapping device has been used. Nevertheless, despite the low number of ions, we observed the effect of the XUV interaction in a moderately large model molecule that is the reserpine, as well as in two large proteins (cytochrome c and insulin). In all these examples, the XUV radiation induces dissociative ionization, meaning that the interaction leads to an efficient valence ionization of the molecule followed by its fragmentation by loss of neutral groups. The advantage of this table-top apparatus is to give access to high energy photons, usually accessible at large scale facility such as a synchrotron. It could cover a large energy range, including soft X-ray. Because the ions are not trapped, it is also possible to include not only mass spectrometers but other types of spectroscopy such as electron kinetic energy measurements. For that goal, the combination of ESI–MS and velocity map imaging^56^ would provide detailed information on structural and dynamical information and could be implemented here in combination with the XUV source. Nevertheless, the current design is not fully appropriate for such implementation. For instance, one would need to extract the ions of interest from argon background and nearby electrodes and transport them to a free interaction region where an electron spectrometer could be installed. Such experiment can be performed even with closely situated rf fields, as demonstrated by the authors^57^. Because of the short pulse duration, time-resolved experiments down to the femtosecond and even attosecond timescale could be performed^34^. This type of experiments offer new perspectives for the development of the emerging field of attochemistry, as well as for analytical purpose where new activation methods are under development. Exciting experiments combining ion storage rings and HHG based XUV sources have been proposed to probe QED effects in heavy atomic ions^58^. Overall, HHG XUV light and ionic species offer a perfect playground to test fundamental physics and chemistry, which should foster further developments.

## Data Availability

The data that support the findings of this study are available from the corresponding author upon reasonable request.
